# Biomechanical Analysis of a Novel Acetabulum Reconstruction Technique with Acetabulum Reconstruction Cage and Threaded Rods after Type II Pelvic Resections

**DOI:** 10.1155/2016/8627023

**Published:** 2016-05-31

**Authors:** Vivek Ajit Singh, Hassan Elbahri, Rukmanikanthan Shanmugam

**Affiliations:** ^1^Department of Orthopaedic Surgery, University of Malaya Medical Centre, 59100 Kuala Lumpur, Malaysia; ^2^Khartoum Teaching Hospital, 11111 Khartoum, Sudan

## Abstract

*Background*. Periacetabular resections with reconstruction has high rates of complications due to the complexity of the reconstruction. We have improvised a novel technique of reconstruction for type II and type II + III pelvic resections with the use of a commercially available acetabulum reconstruction cage (gap II, Stryker) and threaded rods.* Objectives*. The aim of our study is to determine the biomechanical strength of our reconstruction compared to the traditional cemented total hip replacement (THR) designs in normal acetabulum and establish its mode of failure.* Methods*. Five sets of hemipelvises were biomechanically tested (Instron® 3848, MA, USA). These constructs were subjected to cyclic loading and load to failure.* Results*. The reconstructed acetabulum was stiffer and required a higher load to failure compared to the intact pelvis with a standard THR. The mean stiffness of the reconstructed pelvis was 1738.6 ± 200.3 Nmm^−1^ compared to the intact pelvis, which was 911.4 ± 172.7 Nmm^−1^ (*P* value = 0.01). The mean load to failure for the standard acetabular cup construct was 3297.3 ± 117.7 N while that of the reconstructed pelvis with the acetabulum cage and threaded rods was 4863.8 ± 7.0 N.* Conclusion*. Reconstruction of the pelvis with an acetabular reconstruction cage and threaded rods is a biomechanical viable option.

## 1. Introduction

Primary sarcomas of the pelvis account for 10% to 15% of primary bone tumours and pelvis is considered the third most common site for metastasis. Enneking and Dunham had classified pelvic resection depending on the site of the tumour. Tumour involving ilium is classified as type I, tumours involving the periacetabular area as type II, and pubis as type III [1]. Periacetabular bone tumours are considered the most challenging site of all pelvic tumours to treat as they involve the hip joint. Therefore, it is difficult to achieve a good oncological and functional outcome. Periacetabular resections are associated with a high incidence of complications, mainly infection and reconstruction failure [2–4]. This is usually due to the difficulty of reconstruction of the acetabulum cup and the local forces acting on this anatomical region. Type II pelvic resection with wide margin and reconstruction of the soft tissue and bony defect to restore weight bearing along anatomic axes is considered the goal standard management of periacetabular tumours [5, 6]. Many options are available for reconstruction of the bony defect after resections. These include autograft, allograft, allograft composite, and endoprosthesis [7–9]. Endoprostheses are developed to give the most durable and least complicated construct that can cope with the complex anatomy and biomechanical demands of the hip joint [10]. There are various custom-made endoprosthesis available but these are expensive and their long term results are not encouraging. They are associated with high rate of morbidity, infection, and failures [11]. Therefore, we have devised our own method of reconstruction for the acetabular defect using commercially available acetabular reconstruction cage (gap II acetabular cage; Stryker Howmedica), threaded rods, and cemented flanged acetabular cup. We believe that our method of reconstruction for periacetabular tumours might offer a cheaper and easily available alternative compared to other forms of endoprosthesis reconstruction.

**Figure 1:**
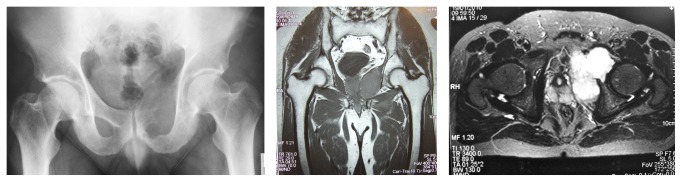
Grade 2 Chondrosarcoma arising from the left superior pubic rami extending into the medial wall of the acetabulum.

**Figure 2:**
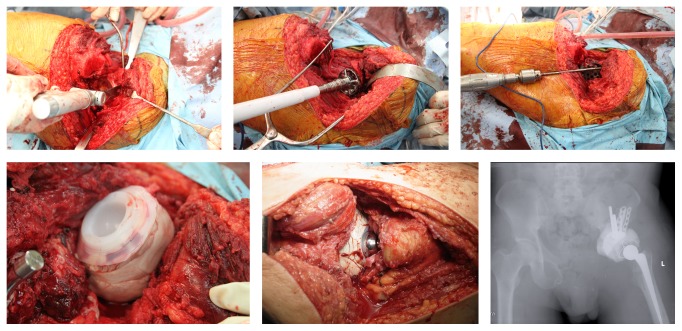
Showing the type II + III resection with acetabulum reconstruction with a acetabulum reconstruction cage and threaded rods.

The example of this form of reconstruction is shown in Figures 1 and 2. This is a 42-year-old gentleman who presented with Chondrosarcoma grade 2 of the left superior pubic rami extending to the medial wall of the acetabulum. He underwent a wide resection and reconstruction with an acetabulum reconstruction cage and threaded rods.

In order to determine the biomechanical strength of this construct, we compared the biomechanical properties of this construct to the standard cemented acetabular component of the total hip replacement.

## 2. Objective

The objective is as follows:

- To determine the biomechanical strength of acetabulum reconstruction cage and threaded rods reconstruction after a combined type II + III pelvic resection.
- To compare the strength of the acetabular reconstruction using acetabulum reconstruction cage and threaded rods in type II and III pelvic resection against the traditional cemented cup in a normal acetabulum.
- To determine the mode of failure of the acetabular reconstruction cage and threaded rods reconstruction designs.

## 3. Materials and Methods

**Figure 3:**
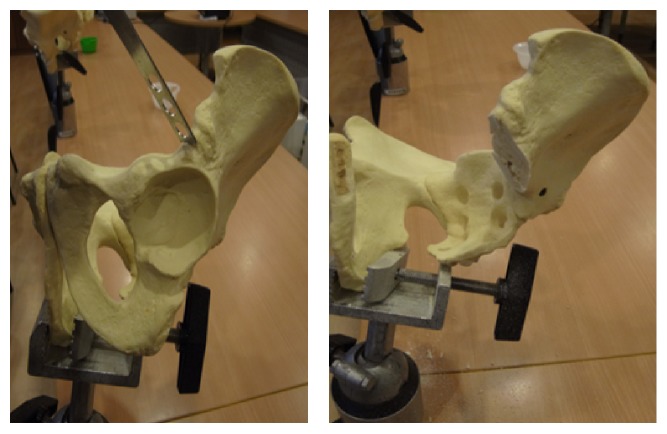
Resection of the pelvis at the level of the supra-acetabulum up to the symphysis pubis to mimic a type II + III pelvic resection.

**Figure 4:**
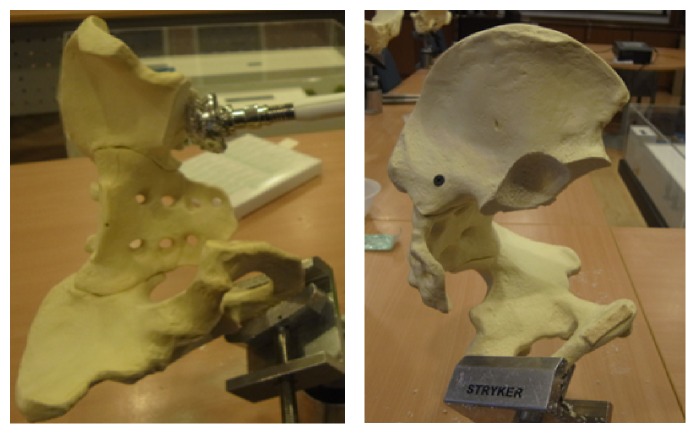
The reaming of the inferior border of the remnant iliac bone to create an indentation for better placement of the gap 2 acetabulum cage.

**Figure 5:**
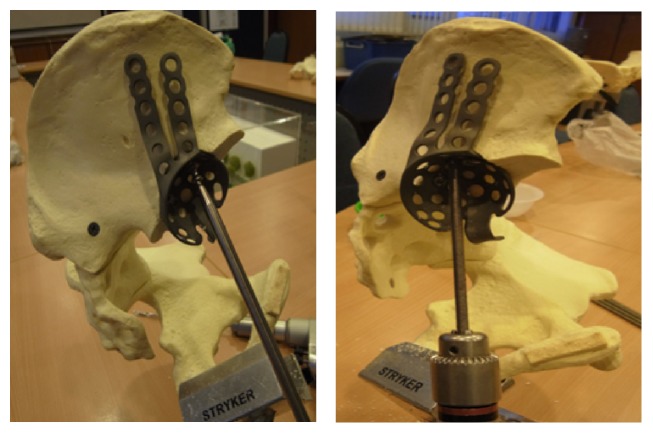
Insertion of long titanium screws and threaded rods to secure the construct.

**Figure 6:**
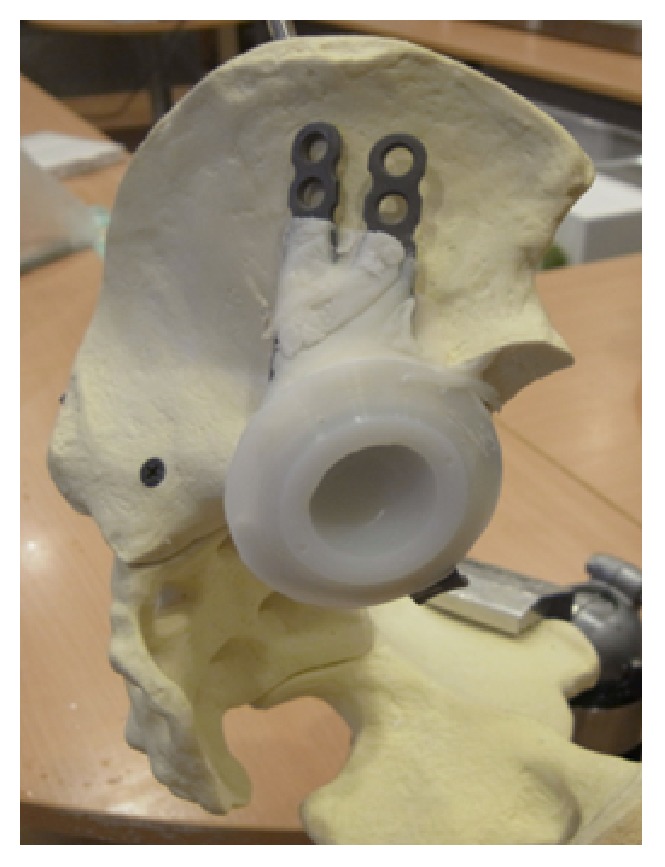
The end product of the reconstruction with a standard flanged cup cemented into the reconstructed acetabulum cage.

Mechanical testing comparing intact pelvis implanted with the standard acetabular cup with those using the modified acetabular reconstruction cage and threaded rods was performed using solid foam bone models of large male full pelvis sourced from SAWBONES. This was to ensure a uniform material property to enable the fixation technique to be assessed with minimal bias. A total of five whole pelvises were used. Five hemipelvises were implanted with a standard acetabular cup (flanged cemented cup from Stryker Howmedica, Group A) according to the manufacturers recommended technique. And in the remaining five, the acetabulum including the pubic bone was resected to simulate a combined type II + III pelvic resection (Figure 3). The inferior border of the remnant ilium is then reamed with the standard acetabulum reamers to create a curve indentation for better placement of the acetabular cage (Figure 4). A gap II acetabular reconstruction cage from Stryker Howmedica is then fitted to the remnant ilium and secured with long titanium screws (Figure 5) in the desired position (45 degrees' inclination and 15 degrees' anteversion) and construct is reinforced with 3 threaded rods. The rods are inserted in the thickest part of the pelvis, consisting of anterior and posterior borders of the ilium towards the anterior and posterior iliac spines and the centre in line with the iliac tubercle (Figure 6). This is known as Group B.

For the biomechanical testing, to ensure uniform loading conditions, the pelvis was positioned on a custom-made jig that would allow the acetabular cup to be placed in the same angle in relation to the femoral stem with a metal head. This was achieved by placing the Anterior Superior Iliac Spine (ASIS) and the anterior part of the pubic tubercle in the same plane and the posterior medial pubic tubercle and the sacroiliac joint surface on another plane which are at right angles to each other. By positioning it in this position, each intact pelvis was able to be placed within the jig and thus the femoral head component in the same position which represents the anatomical position in the natural hip.

**Figure 7:**
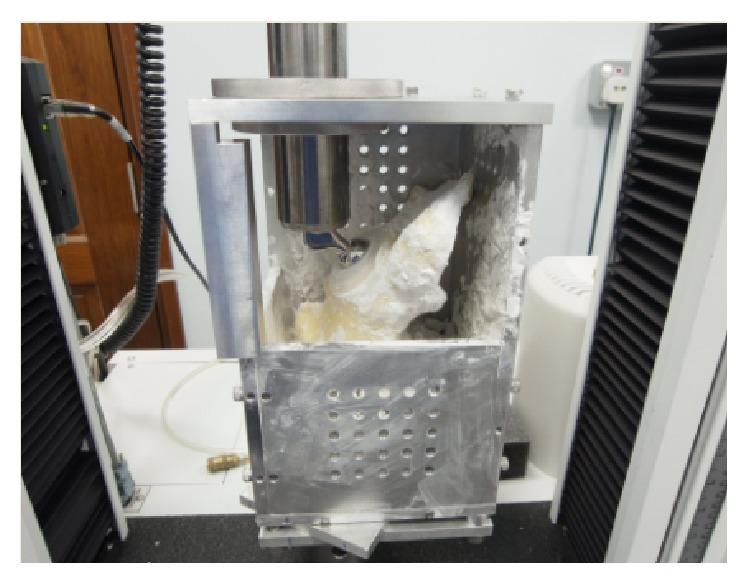
Placement of the test specimen in the test jig which is secured by plaster of Paris in an anatomical position.

The reconstructed specimens (Group B) were positioned in the similar position but since the pubic tubercle is absent, the angle of the acetabular rim in relation to the horizontal and the two vertical planes was measured based on the standard (Group A) specimens thus ensuring a comparable positioning of the reconstructed specimens. This ensured that the force vectors acting on the acetabular cup are consistent for all specimens. The jig used is shown in Figure 7. The specimens were stabilised in the jig by using plaster of Paris.

**Figure 8:**
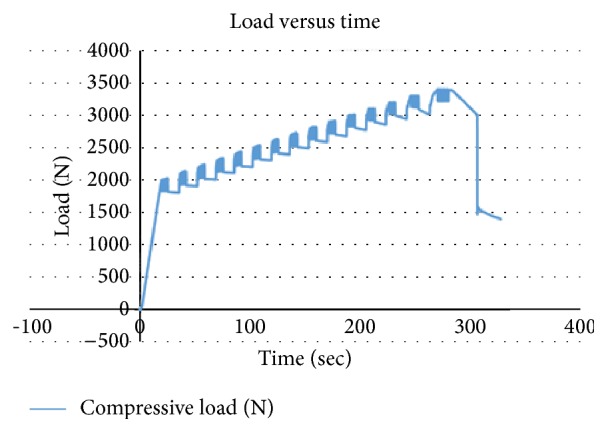
The loading regime for the test specimens.

**Figure 9:**
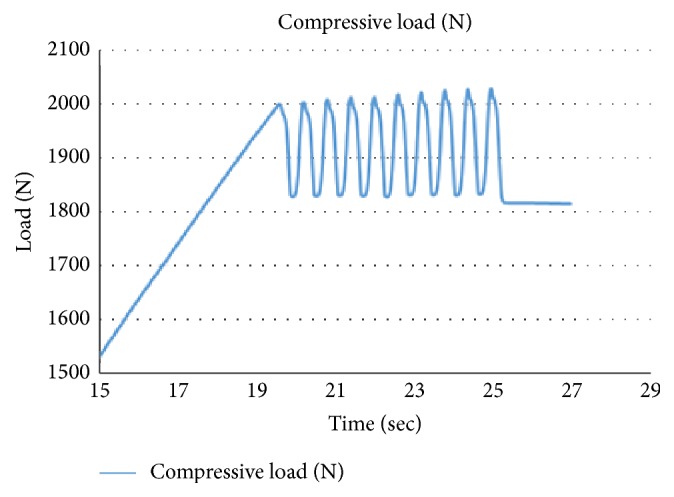
Full loading pattern of the test specimen.

Loading was done using a material testing machine (Instron® 3848, MA, USA) with a 5 kN load cell. A preload of 10 N was applied to take up the slack on the jigs and set the femoral head component well into the acetabulum. A fast ramp of 2000 N was applied with a ramp speed of 0.1 mm/sec. Once 2000 N was reached, load was cycled between 1800 N and 2000 N at a frequency of 1 Hz for 10 cycles. Following this, the 1800 N was held for 10 secs and the load subsequently increased by 100 N and cycled another 10 times. This cycle pattern was repeated until failure. A sample of the overall loading regime is as shown in Figure 8. And a full cycle pattern is shown in Figure 9. Loading was done till failure which was defined as either fracture of the acetabulum or the bony pelvis or displacement of the ramp by more than 20 mm.

The initial ramp was used to calculate the stiffness of the construct which was calculated from the slope of the linear portion of the load displacement curve. The maximum load to failure was also measured for each sample. Data analysis and statistical calculations were done using Microsoft Excel 2010. With the high degree of blocking that was done considering the use of standardised foam models with relatively consistent density and a standardised test setup, *t*-test was done to test for significance using the same software.

## 4. Results

**Figure 10:**
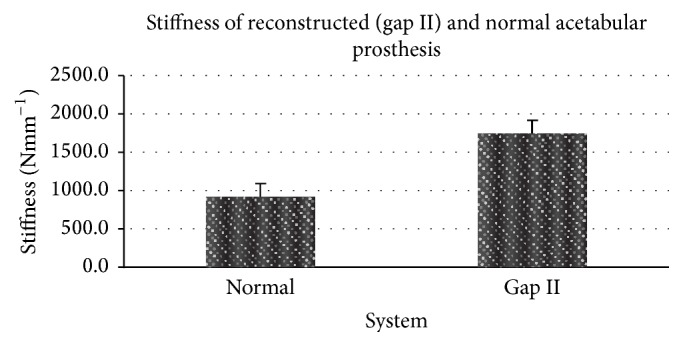
Bar graph showing the stiffness of the reconstructed pelvic and normal acetabular prosthesis.

A total of five full male pelvis sawbones were used. The acetabular reconstruction cage and threaded rods novel reconstruction technique was carried out on half of five hemipelvises and a standard acetabular replacement was applied on the rest of the five hemipelvis sawbones. The reconstructed acetabulum (Group B) was generally stiffer and needed a higher load to failure compared to the intact pelvis with a standard total hip replacement. The mean stiffness of the reconstructed pelvis was 1738.6 ± 200.3 Nmm^−1^ compared to the intact pelvis (Group A) which was 911.4 ± 172.7 Nmm^−1^ (Figure 10). This was statistically significant with a *P* value of 0.01. The mean load to failure for the standard acetabular cup construct was 3297.3 ± 117.7 N while that of the reconstructed pelvis was 4863.8 ± 7.0 N.

All normal pelvis failed by fracture through the iliac bone just above the cemented acetabular cup. Due to the limitation of the testing machine used, none of the reconstructed pelvises could be loaded to failure as the maximum load that can be applied by the machine was reached before failure occurred (all operated specimens were loaded till about 4900 N without failure).

## 5. Discussion

The common sarcomas involving the pelvis are Chondrosarcoma, Ewing's sarcoma, and osteosarcoma [12–14]. These tumours generally present late especially if they are located within the inner table of the pelvis and can grow up to a significant size before becoming clinically apparent [15].

The management of these tumours has always been challenging especially when it involves the pelvis. These surgeries are technically demanding due to the complex anatomy of the pelvis, in particular the acetabulum and hip joint (periacetabulum region) [2, 4, 16–18]. The goals of oncology surgery are the following: first is to get total clearance of the tumour with clear margins and second is to reconstruct the defect to enable best possible function with minimal functional deficit.

In the past decade, these tumours were managed with hindquarter amputations that gave good surgical clearance but poor functional outcome [4, 19, 20]. With advancements in imaging, adjuvant therapy, surgical planning using image fusion and navigation, better surgical techniques, and implant design, limb salvage surgery is possible in these cases [21–27].

Ennenking and Dunham [1] classified pelvic resections according to different regions centred around the acetabulum. In type II and type II + III resections, the acetabulum is removed, therefore, directly affecting the hip joint. Reconstruction options around the acetabulum generally involve fusion of the hip or recreating a new artificial hip joint. Reconstruction techniques without the use of prosthesis include iliofemoral arthrodesis or pseudarthrosis, ischiofemoral arthrodesis, massive allograft, and autoclaved autograft [27]. The common pelvic prosthesis used is the saddle prosthesis [2, 3, 25, 26] but it is associated with a high complication rate. There are numerous other prostheses that have been used and majority of them are custom-made [11, 16, 21, 23, 24, 27]. The Birmingham group recently also reported encouraging results with the use of an ice cream cone prosthesis [8]. Gou from China reported his experience with the use of their own version of a modular pelvic prosthesis that can be used off the shelf without customization [28, 29].

These prostheses are expensive and the majority are custom-made; therefore it takes time (a few weeks) for the product to be ordered, manufactured, shifted, and available for surgery. Sometimes during the wait, the preordered prosthesis is no longer suitable due to tumour progression. Furthermore, as they are custom-made, they are less forgiving to surgical errors made during tumour resections. Hence, we needed a cheaper and a surgical more forgiving alternative that suited our local needs. We decided to use an acetabular reconstruction cage, fortified by insertion of threaded rods into the remaining ilium bone and fixed together with bone cement. As this is a commercially available implant, the cost is much lower compared to a custom-made or a modular pelvic prosthesis. And it allowed us flexibility in terms of tumour progression and additional bone resection intraoperatively.

In order to determine the strength of this new construct, we decided to put it through some biomechanical testing. We compared the reconstructed pelvis to an acetabulum cup replacement in an intact pelvis. We found that the reconstructed pelvis with the acetabular cup was stiffer and required a bigger load to fail compared to a standard acetabulum cup replacement on an intact pelvis.

However, this is a cement bone construct and there is no avenue for bone in growth. Therefore, the long term survivorship of this construct is questionable as compared to other forms of implants that allow osteointegration. Furthermore, the increased stiffness of this construct might predispose this implant to long term fatigue failure especially at the bone cement interface.

Limitation of this study is that we used sawbones to compare the biomechanical strength of each construct. We will require a follow-up study using cadaveric pelvic bones which will give better representation of the biomechanical strength of the construct.

## 6. Conclusion

Acetabulum reconstruction with an acetabulum reconstruction cage and threaded rods after a type II or a type II + III resection is a biomechanically feasible option. The is a cheaper alternative and allows resection flexibility compared to other custom-made pelvic prostheses.
